# A phenotypic drug discovery approach by latent interaction in deep learning

**DOI:** 10.1098/rsos.240720

**Published:** 2024-10-23

**Authors:** Tat Wai Billy Yu

**Affiliations:** ^1^ Macao Polytechnic University, Macau SAR, People’s Republic of China

**Keywords:** drug design, target-agnostic, binding sites, deep learning, latent interaction, phenotypic approach

## Abstract

Contemporary drug discovery paradigms rely heavily on binding assays about the bio-physicochemical processes. However, this dominant approach suffers from overlooked higher-order interactions arising from the intricacies of molecular mechanisms, such as those involving *cis*-regulatory elements. It introduces potential impairments and restrains the potential development of computational methods. To address this limitation, I developed a deep learning model that leverages an end-to-end approach, relying exclusively on therapeutic information about drugs. By transforming textual representations of drug and virus genetic information into high-dimensional latent representations, this method evades the challenges arising from insufficient information about binding specificities. Its strengths lie in its ability to implicitly consider complexities such as epistasis and chemical–genetic interactions, and to handle the pervasive challenge of data scarcity. Through various modeling skills and data augmentation techniques, the proposed model demonstrates outstanding performance in out-of-sample validations, even in scenarios with unknown complex interactions. Furthermore, the study highlights the importance of chemical diversity for model training. While the method showcases the feasibility of deep learning in data-scarce scenarios, it reveals a promising alternative for drug discovery in situations where knowledge of underlying mechanisms is limited.

## 1. Introduction

In drug development, deep learning has captivated the attention of researchers. From predicting intricate drug–target interactions to evaluating drug–drug similarities and beyond, the applications of deep learning present exciting frontiers in the quest for more efficient and effective pharmaceutical advancements [1–3]. Mostly, it involves exploring the chemical and molecular spaces with approaches like homology for potential drug candidates [4]. The advantages are speed and interoperability [5]. However, beneath it lies a critical challenge: the need for reliable patterns in docking specificity, structural or functional properties like in spike protein, pharmacological perturbation, and transcriptomic signature matching [6–9]. The realization of docking strategy is restrained by the lack of high-quality transcriptome-wide profiling data [10]. Their applications necessitate reliable data from diverse fields such as pharmacological targets [11], physicochemical characteristics [12] and drug pharmacokinetics [13], which are usually costly.

Virus gene sequences now provide a device in rational drug design [14]. However, data sufficiency again becomes a concern. Say, VolSite [15,16] offers promise in this pursuit, yet its development path is not without challenges. Complications such as epistasis [17], off-target effects, traverse regulatory networks, competition for binding [18], the noncoding regions [19], synergistic interactions [20] and the interactome molecular pathways [21] in the host and other bio-physicochemical interactions contribute to snags beyond anticipated bindings [22]. Understanding interactions among multiple loci proves challenging. The nonlinear dynamics within the realm of biocomplexity make it virtually impossible to anticipate all potential higher-order interactions [23,24]. It is also difficult to assay complex phenotypes at scale [25]. Despite employing sound strategies [2,3,5,9,26–30], target-binding methods in clinical drug development are hindered by high false-positive rates [28,31].

As deep learning technology continues to evolve, models to address drug development challenges become more pertinent. To this end, this study aims to harness its transformative power in advancing the methodology. Instead of using discovered patterns in docking specificity, it devises a method that relies exclusively on the therapeutic information of drugs. Hopefully, the automatic feature extraction ability in today’s deep learning can facilitate drug prediction. In essence, this study introduces a deep learning approach to examine the complex interactions that are otherwise impossible in a typical target-based approach.

### 1.1. Current tricks for interaction recognition

Similar unorthodox methods have emerged to bypass typical reliance on explicit binding specificity information in the literature; they only leverage other strategies. A noteworthy example of this is a network-based approach. It taps into the network proximity of drug-gene signatures [32]. Network-based methodologies with phylogenetic analyses can also quickly identify candidate drugs [33]; it exemplified the potency of the network-based approach in the context of coronavirus research. In short, the network-based approach demonstrates a rapid and effective strategy without the need for explicit binding specificity; however, there is room for improvement in its performance on accuracy (the discussion section will elaborate more on this).

Conversely, metabolism functions, molecular mechanisms and a considerable proportion of intracellular proteins are regulated by the genomic sequence through the modulation of gene expression. This paradigm offers a promising avenue for drug repurposing endeavours with notions in the transcriptomics-based pipeline. Through it, deep learning methods can work out solutions by automatic feature extraction. Despite the absence of explicit therapeutic targets in various diseases and biological processes, models can glean insights from both chemical structures and gene expression changes [34]. With extracted latent representation of chemical molecules and landmark genes, researchers markedly demonstrate the ability to predict intricate interactions [2] and, more specifically, binding affinity [17]. These methods exploit the deep learning working mechanisms without full understanding of the underlying molecular processes. This empowers researchers to unravel highly complex bio-physicochemical relationships that cannot be manifested in conventional methods.

Whole-genome analysis has repeatedly demonstrated its clinical facilitation [35]. In microscopic study of pathogens, it increases the power of molecular epidemiology and helps develop novel treatments and vaccines [36]. In macroscopic epidemiological research, it offers a reliable means to assess the extent of viral transmission [37]. With the accessibility of whole-genome sequencing, deep learning methods could transcend the limitations of explicit targets and empower researchers with a more comprehensive approach to understanding biological processes.

### 1.2. Utilizing molecular mechanisms

There are diverse methodologies employed in virus-based treatment options. They focus on viral nucleosides, nucleotides and nucleic acids by leveraging transcriptomics, molecular docking, and the analysis of structural and functional properties [38]. Amidst that, this study introduces a novel approach grounded in logic of the complex interaction among viral genes and drugs. The genetic makeup shapes viruses’ structure, function and behaviour. Some replicate and control the expression, regulate and encode proteins that interact with their host cell proteins. Recognizing this pivotal role of genes, I propose a method to harness genetic information as a comprehensive summary of functional and behavioural features for in-depth deep learning analysis with the drug.

In like manner, the functionality of a drug is shaped by its chemical makeup, which can be represented by the International Chemical Identifier (InChI) [39]. The InChI is a text string that effectively identifies and summarizes the chemical information, including the molecular structure, bonding, isotope, stereochemistry, etc. By recognizing this representation as a source of drug information about its functionality and behaviour, we can utilize deep learning analysis to probe into its interaction with viruses.

However, the challenge lies in the extraction and transformation of the information into meaningful features for modelling the interactions. Traditional docking strategies depend on explicit means by geometric features. In contrast, the strategy here tries to extract features automatically [40] with proper modeling skills in deep learning. A detailed description of our approach to achieving the designated objectives will be presented in §§2.1 and 2.2.

In essence, this novel approach draws an analogy to how our ancestors discovered drugs through the observation of their effects [41]. This phenotypic drug discovery approach has lately been supplanted by the reductionist approach of modulating specific molecular targets of interest [42]. Distinct from the assumptions about specific genome regions, our target-agnostic approach bypasses functional preconceptions of introns, exons, non-coding regions, etc. Also, it makes no assumption about the functionality of specific genome regions. Instead, it uses end-to-end learning to acquire knowledge of molecular targets or even pathways implicitly. §2 altogether serves as a meticulous exposition of our process, wherein deep learning models are wielded to scrutinize complex interactions.

The focus of this study is on leveraging deep learning to unveil specificities, targets or mechanisms in the latent space, in which the latent space analysis approach has shown success in molecular interactions within the tumor microenvironment [43]. This approach harmoniously integrates reductionist concepts with the formidable capabilities of deep learning tools. This amalgamation aims to untangle the interaction intricacy based on the discovered drugs and virus genetic information. The rest of this paper presents the comprehensive strategy.

## 2. Material and methods

I approach the drug–virus problem as a supervised deep learning task. An intention is to recognize potential interactions through end-to-end learning. Nevertheless, deep learning techniques require massive data for model training but there were only 58 antiviral-drug available in the Virus Pathogen Resource (ViPR) [44]. This small sample size does not provide enough therapeutic cues for most deep learning models to learn. Data scarcity is an impediment. In contrast, there are more than ten thousand FDA-approved drugs (10,282). FDA approval is a decent inclusion criterion because the FDA undergoes a rigorous review process to ensure drug eligibility. It should provide guaranteed information and sufficient accessible chemical diversity for a model to learn some pharmacophores [45]. We can utilize this availability. A simple data augmentation technique can translate this data availability into the feasibility of deep learning analysis.

Therefore, I created negative samples using data augmentation [46,47]. Pairs of virus genes and drugs were each labelled with their compatibility, a match or a mismatch. When a drug was approved by FDA for a virus disease, the corresponding pair was considered effective for the treatment; I assigned a value of one (1) to it. For the mismatch, a pair was assigned a value of zero (0 a negative sample). However, this negative assignment might be a misjudgment, resulting in a trained model wrongly disqualifying a drug. In this setup of maximizing chemical diversity, I fabricated negative training examples with minimal repetitions to reduce potential falsely mismatched noises. This was accomplished by a data augmentation technique to enable supervised deep learning models to work on a typical binary prediction problem.

The 58 drugs served as seeds for creating an authentic drug–virus dataset. The number of unique virus names (organism names) was 198. The model aims to predict the drug efficacy based on the textual inputs: nucleotide sequence of the virus and the drug representation. I mapped the virus names to the NCBI Viral Genomes Resource [48]; 775 unique assemblies of viruses were identified. I then obtained the genome sequences from GenBank. The number of unique genome sequences at the nucleotide level in FASTA representation was 1353. Long sequences of this number shall provide enough nucleotide information for the training process.

For the drug part, I used InChI instead of SMILES to represent chemical structure because it is more informative. Although SMILES are more human-readable, the InChI format separates the information about atoms and bonds. It explicitly describes the structure of a molecule and the bonding between atoms; its hierarchical layered line notation further provides information about the structural complexity. Thus, it has an advantage in dealing with complexities like isomers, isotopic distributions and more importantly the type of nomenclature that helps understand the structure of a molecule.

Despite the fact that the InChI representation provides more information about drug molecules, there were successful cases of using the SMILES representation for deep learning model to learn. For SMILES, character strings were successfully encoded by using recurrent neural networks (RNNs) as well as convolutional neural networks (CNNs). Nevertheless, good performance was contributed by the presence of repetitive, translationally invariant substrings that correspond to chemical substructures, e.g. cycles and functional groups [49,50]. On the other hand, the InChI representation has a more complex syntax that includes counting and arithmetic. Its hierarchical layers also hinder the feasibility of analyzing the text strings as plain sequences. Encoders with relatively simple RNNs and CNNs would not be able to perform. InChI layering structure demands more sophisticated deep learning architecture, like the language model, to understand it for prediction accuracy. To solve the problem, I deploy members from the seminal work of the transformer-family [51] to handle its language-like complication and its layering structure. §2.2 is dedicated to the custom-building process.

Data construction took into consideration its uncommonness of drug repurposing and practicability in training. Drugs uncovered for repurposing are rare; the arrangement of unbalanced data is inevitable. However, the rarity shall not be too extreme that it brings unnecessary technical analysis difficulties. Thus, the number of mismatches (negative samples) was roughly 12 times (12 ×) more than the matches. To evaluate model performance, data was partitioned into three (3) pieces: training, validation and testing data. In order to preclude potential data leakage, authentic matches of an antiviral drug were exclusive in only one of the data partitions. §3.1 will reveal more detail. To further evaluate the potential of data leakage, results will be accompained with some drug similarity assessment to examine the reliability.

For the model to learn, the training data were of the largest proportion. It contained 208 223 drug–virus pairs, of which 14 646 (6.9%) were authentic matches. The validation data provides the first test against unseen data, it helps to evaluate how well the model can predict results of new data, oversee the training process of potential overfitting and tune the model. It had 59 424 pairs, with 4104 authentic (6.7%). The test data examined the model prediction accuracy after the model was built. It provided a decisive real-world check of an unseen dataset to substantiate model performance. It contained 30 244 pairs, with 2591 authentic (8.4%) for ultimate out-of-sample evaluation.

**Figure 1.**
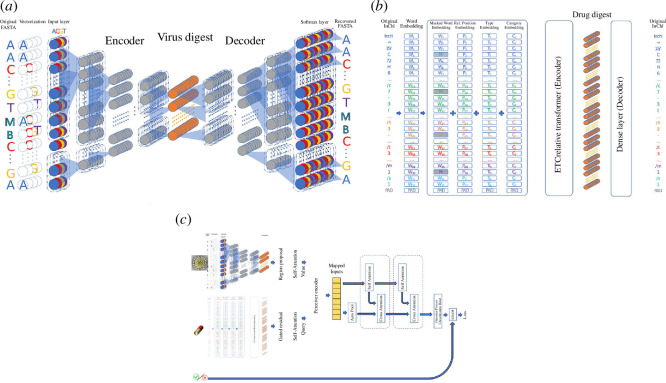
The model framework. Panel (*a*) is a one-dimensional CNN-based ResNet autoencoder for the virus FASTA representation. The resulting virus digest is coloured orange. Panel (*b*) is an autoencoder for the drug InChI representation. The encoder is essentially the ETC model. Panel (*c*) shows the core model. After the stylish self-interaction search for the digest individually, a Perceiver model searches for meaningful interactive features iteratively.

In contrast to conventional methods, the inputs are simply the InChI representations of drugs and the genetic information of viruses. Deep learning models are to convert these text strings into high-dimensional latent data for the process. This approach tackles the issues of scarce data and complex biological and chemical properties. To the best of the author’s knowledge, no previous research took this alternative path of using pure text strings as inputs and language models for the problem. Thus, I made available the datasets for other researchers to work on^1^. Figure 1 illustrates the framework of this study.

I used the ensemble method [52] to formulate and train the model in a modular way. It starts with the basic premise that we should maximize the utilization of available information on both viruses and drugs and we should tickle problems with relevant deep learning architecture. I preprocessed the inputs with pre-trained models to obtain representations before the interaction search. Model training consisted of two phases. The first phase involved learning relevant features of drugs and viruses with two independent autoencoders so that they could extract meaningful representations for reconstruction of corresponding raw inputs. These models leveraged the power of automatic feature extraction to generate drug and virus latent features. They were optimized to preserve essential chemical/genetic information so that the original entities could be reconstructed from the latent features. This phase focused on creating reliable encoding models for drugs and viruses. The encoder parts of the models can produce latent representations for inputs. §§2.1 and 2.2 will detail the process. This technique shall help circumvent the issues of over-parameterization and overfitting, and §3 will substantiate that. The outputs were two encoding models for drugs and viruses. They are responsible for creating latent features as digests. The second phase developed a deep learning classification model that takes the digests as inputs. It searches for interactions concerning the drugs and viruses in the latent space, then predicts the effectiveness of the drug on a virus supervised by the compatibility label (0 or 1). I applied various technical tricks and justified them based on the biochemical or genetic literature. §§2.3 and 2.4 will detail the process.

### 2.1. The virus digest

The first preprocessing module is for virus representation. Non-coding regions may regulate transcription, and its binding may affect the binding site affinity, specificity and diversity [53,54] and related cellular functions [19]. I used an unsupervised learning technique to process the whole genomic sequences to avoid missing out on this representation learning. The encoder module takes the virus genome in the 4-nucleobase form and converts it into a vector. The vector had 0’s or 1’s at the index bases. This input vectorization method allows multiple alternative representations in a nucleotide position, like for M and N.

I used an autoencoder to capture representative information and to reduce the long sequence to a shorter and more manageable length. Figure 1*a* depicts the autoencoder; it consists of two parts: the encoder extracts the latent features of viruses, and the decoder tries to reconstruct the original input sequence. The model learns to minimize the reconstruction errors. I selected the ResNet [55] because it can compress lengthy sequences, and its residual connections make weight updates more effective. This way, more layers could be stacked together for abstract learning. The ResNet model was implemented in eight blocks of process, each block consisting of two sub-blocks of one-dimensional convolution neural network (CNN) and batch normalization. Stages were connected with residual links. Its principle hidden dimension is 128, and it was trained with a batch size of 64. I also used squeeze-and-excitation optimization [56] for performance. It helps feature recalibration and can learn to use global information to select useful features and suppress others. It was proven effective for ResNet, while interdependencies between channels (of nucleotide bases) are unknown but crucial.

### 2.2. The drug digest

Language models, which rely heavily on transformers, can learn representations of large-scale structural characterization from single protein sequences [47]. I leveraged this ability of transformers to prepare the drug pre-trained model. The InChI representation is similar to language and has a hierarchical structure. InChI allows encoding with different levels of granularity (a representation can include or exclude stereochemical, isotopic and tautomeric information [39]). However, with this complex structure of InChI (unlike SMILES), relatively simple RNNs and CNNs would not be able to perform.

Recent development of deep learning in natural language processing (NLP) enables effective ‘understanding’ of such human-readable textual input (for example [57]). Nevertheless, regular transformers in conventional NLP models are less capable of understanding when the text comprises some hierarchical structure; fortunately, the extended transformer construction (ETC) model is designed to handle the challenge of encoding structured text [58]. It can extract meaningful molecular descriptors in the form of latent molecular representations for structures without the need for additional or explicit labels. As regularly practised, I engineered the InChI input into several layers to enhance its understanding about the textual description. As shown in figure 1*b*, the most basic layer was the *word embedding* layer. It tokenizes the chemical elements and other chemical information. Specific to InChI representation, a forward slash followed by a letter signifies the kind of information. For example, it identifies the protonation by '/p' and stereo sublayer by '/s'; this information is stored in the *type embedding* layer. The position embedding is a distinctive design for relative attention in the ETC model. Coupled with its *relative position encoding*, it can effectively capture complex hierarchical structures in the InChI representation. For numeric information, the number token explicitly identifies it and the *category embedding* signifies that it is a number. An advantage of this setting is that it imposes no conflict with the ETC hierarchical attention mechanism. The training of this autoencoder also followed well-regarded randomly masked words pre-training strategy [59], so as to predict the original word based on the context. The ETC model was implemented with six transformer layers, each with two attention heads and model hidden dimension of 128, resulting in a model depth of 64. This is the dimensionality used for the individual attention heads. The inner dimension of the feedforward neural network (FNN) is 512. For the training, the masking proportion was 10% and the batch size was 64.

### 2.3. Self-association

Before examining drug–virus interactions, I developed a scheme to handle potential self-association in the analysis context. For the gene part, the scheme considers the functional nucleotide motifs, regulatory sequences, self-complementary sequences, secondary structures, etc. Non-coding regions may also affect gene expression by binding to transcription factors, resulting in the activation or repression of transcription. The scheme should consider all of the above at a genome-wide scale, but without the conventional binding and gene expression information. To detect object bounds and objectness, I applied an object detection method with the region proposal network (RPN) [60]. This proven technique works well with the attention mechanisms that we will use in the cross-interaction search in the subsequent analysis phase. For the RPN, there were two layers of one-dimensional convolution networks, both with hidden dimensions of 128. The first layer was of kernel size of 3 and the other of 1, in between them was a unified (batch) normalization and (ReLU) activation layer. After this were two self-attention layers with hidden dimension of 128 in both the query and the value channels. Figure 1*c* shows the schematics of the analysis.

Similar self-association is also present in the drug part. It can be the modulation of physicochemical properties or the mechanisms of drug solubilization [61]. The InChI representation input is multifarious; without prior information on possible self-interaction, I adopted a gating mechanism to skip over unused components [62]. It enables high performance in a wide range of scenarios for unused parts and provides efficiency and flexibility to apply non-linear processing only where needed. The implementation of this gated residual network followed the standard, the first layer was a linear FNN. The second was a simple activation layer with an exponential linear unit (ELU). The third layer was again a linear FNN. The last was a so-called gating layer made up of two time distributed wrapped dense layers and multiplying layers with a dropout rate of 15%. All intermediate layers had hidden dimensions of 512 and the output dimension was 128.

In the context of the drug–virus interactions, *cis*-regulatory elements, such as promoters, enhancers and silencers, regulate the transcription of nearby genes and are likely to complicate the biochemical processes. Both local and global contexts should matter for modelling dependencies among inputs. A deep learning technique strives to address this complex combinatorial scenario. Inspired by estimating interactions between pairs of gene features in studying regulatory DNA sequences [63], I utilized context-aware self-attention [64]. This self-attention is a proven technique to capture local and global contexts. This scheme efficaciously probed potential self-interactions of modeling both short- and long-term dependencies. Figure 1*c* shows the schematics of using context-aware self-attention.

### 2.4. Cross-interaction search

Putative drug–gene interactions only indicate binding potentials; they do not always guarantee drug effectiveness [65]. Similarly, the absence of interactions at certain gene loci or codons does not imply that they are irrelevant to drug efficacy. Other factors may also complicate the bio-physicochemical interactions besides the binding specificities. For example, a drug can affect gene expression levels [66,67], and there are also issues related to overlooked exons, hidden binding pockets [68], functions of recoded stop codons or regulators [69] and structural entanglement factors [70]. The combinatorial relationship of the binding specificities is difficult to capture. I adopted the Perceiver mechanism [71] to learn higher-order interactions based on the digests and preprocesses that contain high-level drug and gene self-associations in the latent space, figure 1*c*. It alternated between cross-attention and self-attention to process the mapped inputs and efficiently capture possible combinations. It hence identifies meaningful interactive features. More specifically, it aims to determine the effects of pharmacogenetic polymorphisms in the latent space by iteratively attending to all latent features. The perceiver encoder took the virus’s part as the value and the drug’s part as the query. It was made up of four self-attention layers, each of two attention heads and of hidden dimension of 128, which means a depth of 64. The pre-processed resultant was the mapped inputs. Afterward, the cross-attention and self-attention units were implemented with two attention heads and a hidden dimension of 128, that is again a depth of 64.

Since there were no labelled segments of genes and drugs in the interaction, I used an adaptive pooling layer, AutoPool, to preserve the temporally dynamic predictions in the pooling process. This modification is a proven technique to detect weakly labelled sound annotation [72], which is a characteristic that fits our iterative search for latent features in the sparse and unknown interaction in long sequences. Another minor but technically important issue was that the data contained observations distant from one another, especially with the mismatches of drugs. A model should be aware of the magnitudes of the differences between the current samples and the previously seen training examples [73]. Bayesian approaches would be the best candidate to handle such uncertainties; however, they require another framework on Bayesian neural networks. That would demand excessive computational and memory requirements. For performance concerns, I used a Gaussian Process Classification Head based on the spectral-normalized neural Gaussian process (SNGP) [74]. It introduced a simple but high-quality uncertainty estimation method to handle the classification problem by adding a spectral normalization during training.

Deep learning models may not learn efficiently from class-imbalance data. To ensure that the training process was not affected by distribution variability, I replaced the standard cross-entropy with a label-distribution-aware margin (LDAM) loss in the training process [75]. It improves the generalization error of minority classes without sacrificing the ability to fit the frequent classes. I also incorporated label smoothing [76]. This regularization technique turns deterministic class labels into probability distributions in the model training phase. It is a popular technique to improve performance on class-imbalance data. Furthermore, multiple performance metrics were used for evaluations. The appendix details their statistical significance.

## 3. Results

**Figure 2.**
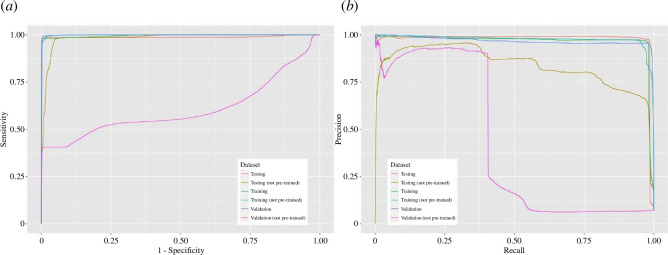
Model performance curves. Panel (*a*) presents the receiver operating characteristic (ROC) curves, which show how sensitivity (TPR) and sensitivity (PR) vary with the threshold. Panel (*b*) presents the precision–recall (PR) curves, which examine the model’s ability to find those minority observations.

The proposed model’s overall results are shown in the top row of table 1. The area under the receiver operating characteristic curve (AUC = 99.5% for the training and 98.8% for the test data partition) is high. However, AUC focuses on the relation between the true positive rate and false positive rate; a good performance might present an optimistic picture because true negatives dominate, for the unbalanced datasets that we have. Figure 2 presents both the receiver operating characteristic (ROC) and precision–recall (PR) curves side-by-side; they together scrutinize the model’s effectiveness and strength. The colour of the lines indicates the data partitions and models that they represent. As anticipated, both models, with and without pre-training, perform well on the training data partition (the green and the light blue lines in both the ROC and PR curves).

**Table 1.** Model performance and assessment of the refinements. Evaluations show performance degradation in the absence of specific refinements.

purpose	trick	alternative	data partition	AUC	accuracy	precision	recall	F1 score	MCC
full model with all the tricks to handle relevant issues	including all the tricks listed below	—	training data	99.5%	99.6%	95.9%	98.0%	0.969	0.967
			validation data	99.8%	99.5%	95.4%	98.0%	0.967	0.964
			test data	98.8%	99.6%	96.9%	98.5%	0.977	0.975
representative and effective feature extraction	pretraining with autoencoders	encoder not pretrained	training data	99.9%	99.3%	93.3%	96.5%	0.949	0.945
			validation data	60.5%	95.4%	84.5%	40.4%	0.547	0.566
			test data	98.3%	94.4%	87.8%	40.9%	0.558	0.577
object bounds and objectness in genes	region proposal network	absence of the trick	training data	96.5%	99.1%	93.0%	93.6%	0.933	0.928
			validation data	87.0%	97.9%	94.2%	74.3%	0.831	0.827
			test data	97.4%	99.3%	96.2%	95.1%	0.957	0.953
skipping over unused components in drugs	gating mechanism	absence of the trick	training data	98.5%	99.5%	95.4%	97.4%	0.964	0.961
			validation data	93.1%	98.8%	95.4%	86.4%	0.907	0.901
			test data	98.8%	99.5%	96.5%	98.0%	0.973	0.970
capturing local and global contexts	self-attention	absence of the trick	training data	98.8%	99.6%	96.4%	97.9%	0.972	0.969
			validation data	74.6%	95.9%	84.7%	49.9%	0.628	0.631
			test data	89.2%	97.5%	90.4%	79.2%	0.844	0.833
temporally dynamic predictions	adaptive pooling layer	absence of the trick	training data	98.4%	99.5%	96.2%	97.1%	0.967	0.964
			validation data	89.6%	98.4%	97.0%	79.4%	0.873	0.869
			Test data	98.7%	99.6%	97.7%	97.7%	0.977	0.975
uncertainty estimation	gaussian process classification head (SNGP)	absence of the trick	training data	96.9%	99.2%	93.8%	94.2%	0.940	0.935
			validation data	96.0%	99.1%	93.9%	92.5%	0.932	0.927
			test data	99.0%	99.4%	95.1%	98.5%	0.968	0.965
generalization error of minority classes, with the ability to fit the frequent class	label-distribution-aware margin (LDAM)	standard cross-entropy	training data	98.3%	99.5%	95.7%	96.9%	0.963	0.960
			validation data	92.1%	98.7%	95.5%	84.5%	0.897	0.892
			test data	99.1%	99.6%	96.3%	98.6%	0.974	0.972
regularization for class-imbalance data	label smoothing	no label smoothing	training data	97.1%	99.3%	95.5%	94.6%	0.950	0.947
			validation data	91.2%	98.5%	95.6%	82.7%	0.887	0.882
			test data	97.6%	99.3%	96.7%	95.5%	0.961	0.958

Nevertheless, while deep learning models are prone to overfitting, we need to assess the performance of other data partitions. For instance, the model without pre-training seems to perform well on the testing data (the yellow line on sensitivity, figure 2*a*). However, its PR curve (the yellow line in figure 2*b*), which accounts for the potential inflation in the false positive rate (FPR), shows that the good performance on ROC is a trade-off. It just incorrectly generated more positive predictive values. Thus, the PR curves help us evaluate the models’ ability to correctly predict the minority, which are the authentic matches. The overfitting phenomenon is more noticeable in the performance for validation data, which contains a greater number of observations for evaluation. Both the ROC and PR curves (the violet lines) show that the model without pretraining cannot perform as well as the model with pretraining (the blue lines). After all, by comparing the areas under the curves, both ROC and PR curves agree that the model with pre-trained encoders performed better. To illustrate the prediction of drug-virus interaction concerning these two models, a electronic supplementary material, table S1, was created. It shows that, for the proposed model, the true positive rate is high (over 98.0% for all datasets), yet the true negative rate is even higher (99.7% for all datasets). The model is more capable in scrutinizing and disqualifying a drug than in confirming its effectiveness. On the other extreme, it illustrates that an alternative model, which was without pre-trained encoder, was not able to rediscover a drug’s effectiveness but was only good at disqualifying a drug when it was with an irrelevant virus.

Looking into the prediction ability for drug discovery, table 2 presents the subset result on FDA-approved antiviral drugs. While authentic matches of a drug were exclusive in only one of the data partitions, the model correctly rediscovered 29 out of 40 approved antiviral drugs in the training data, shown in the upper half of table 2. The remaining 11 drugs had too few observations for the model to learn in the training process: Idoxuridine, Penciclovir, Trifluridine and Valaciclovir had only four authentic matches each. Famciclovir and Aciclovir had 6; Foscarnet had 8; Vidarabine had 10; Entecavir, Adefovir Dipivoxil and Telbivudine had 35 matches each. For the 11 drugs in the validation data, the trained model correctly rediscovered nine of them. For the seven authentic drugs in the test data, the trained model correctly rediscovered six of them, missing Valganciclovir. Apart from those unsuccessful cases, most recall values were high (over 0.93), except for Boceprevir, Ledipasvir and Ombitasvir. These three drugs might share some but not all bio-physicochemical characteristics or molecular pathology with other drugs in the training data.

**Table 2.** Prediction performance on approved antiviral drugs. Model prediction result for FDA-approved antiviral drugs. There are many NAs (represented by a hyphen) in which the prediction has no true positive (TP).

	drug		training dataset					validation dataset					testing dataset				
			accuracy	precision	recall	F1 score	MCC	accuracy	precision	recall	F1 ccore	MCC	accuracy	precision	recall	F1 score	MCC
training dataset	DB00194	Vidarabine	0.998	—	0.000	0.000	—	1.000	—	—	—	—	1.000	—	—	—	—
	DB00198	Oseltamivir	0.994	0.974	1.000	0.987	0.983	0.996	0.000	—	0.000	—	0.988	0.000	—	0.000	—
	DB00220	Nelfinavir	0.991	0.966	1.000	0.983	0.977	0.996	0.000	—	0.000	—	0.995	0.000	—	0.000	—
	DB00224	Indinavir	0.993	0.970	1.000	0.985	0.980	0.998	0.000	—	0.000	—	1.000	—	—	—	—
	DB00249	Idoxuridine	0.999	—	0.000	0.000	—	1.000	—	—	—	—	1.000	—	—	—	—
	DB00299	Penciclovir	0.999	—	0.000	0.000	—	1.000	—	—	—	—	1.000	—	—	—	—
	DB00300	Tenofovir disoproxil	0.992	0.968	1.000	0.984	0.979	0.984	0.000	—	0.000	—	0.990	0.000	—	0.000	—
	DB00426	Famciclovir	0.999	—	0.000	0.000	—	1.000	—	—	—	—	1.000	—	—	—	—
	DB00432	Trifluridine	0.999	—	0.000	0.000	—	1.000	—	—	—	—	1.000	—	—	—	—
	DB00442	Entecavir	0.992	—	0.000	0.000	—	1.000	—	—	—	—	1.000	—	—	—	—
	DB00478	Rimantadine	0.994	0.977	1.000	0.988	0.985	0.991	0.000	—	0.000	—	0.996	0.000	—	0.000	—
	DB00503	Ritonavir	0.992	0.966	1.000	0.983	0.978	0.995	0.000	—	0.000	—	0.986	0.000	—	0.000	—
	DB00529	Foscarnet	0.998	—	0.000	0.000	—	1.000	—	—	—	—	1.000	—	—	—	—
	DB00558	Zanamivir	0.999	0.998	0.998	0.998	0.997	0.999	0.000	—	0.000	—	0.998	0.000	—	0.000	—
	DB00577	Valaciclovir	0.999	—	0.000	0.000	—	1.000	—	—	—	—	1.000	—	—	—	—
	DB00649	Stavudine	0.991	0.964	1.000	0.982	0.976	0.988	0.000	—	0.000	—	0.986	0.000	—	0.000	—
	DB00718	Adefovir dipivoxil	0.992	—	0.000	0.000	—	1.000	—	—	—	—	1.000	—	—	—	—
	DB00787	Aciclovir	0.999	—	0.000	0.000	—	1.000	—	—	—	—	1.000	—	—	—	—
	DB00811	Ribavirin	0.976	0.981	0.931	0.955	0.940	0.993	0.000	—	0.000	—	0.993	0.000	—	0.000	—
	DB00879	Emtricitabine	0.988	0.955	1.000	0.977	0.970	0.993	0.000	—	0.000	—	0.991	0.000	—	0.000	—
	DB00915	Amantadine	0.989	0.957	1.000	0.978	0.971	0.975	0.000	—	0.000	—	0.991	0.000	—	0.000	—
	DB00943	Zalcitabine	0.981	0.926	1.000	0.962	0.950	0.981	0.000	—	0.000	—	0.967	0.000	—	0.000	—
	DB01048	Abacavir	0.985	0.940	1.000	0.969	0.960	0.990	0.000	—	0.000	—	0.987	0.000	—	0.000	—
	DB01232	Saquinavir	0.991	0.964	1.000	0.982	0.976	0.978	0.000	—	0.000	—	0.990	0.000	—	0.000	—
	DB01264	Darunavir	0.997	0.989	1.000	0.994	0.992	0.998	0.000	—	0.000	—	1.000	—	—	—	—
	DB01265	Telbivudine	0.992	—	0.000	0.000	—	1.000	—	—	—	—	1.000	—	—	—	—
	DB01319	Fosamprenavir	0.992	0.968	1.000	0.984	0.979	0.980	0.000	—	0.000	—	0.991	0.000	—	0.000	—
	DB01601	Lopinavir	0.993	0.972	1.000	0.986	0.981	0.993	0.000	—	0.000	—	0.995	0.000	—	0.000	—
	DB05521	Telaprevir	0.999	1.000	0.960	0.980	0.979	1.000	—	—	—	—	1.000	—	—	—	—
	DB06414	Etravirine	0.990	0.964	0.998	0.981	0.975	0.991	0.000	—	0.000	—	0.981	0.000	—	0.000	—
	DB06817	Raltegravir	0.989	0.959	1.000	0.979	0.972	0.985	0.000	—	0.000	—	0.996	0.000	—	0.000	—
	DB08864	Rilpivirine	0.990	0.959	1.000	0.979	0.972	0.986	0.000	—	0.000	—	0.991	0.000	—	0.000	—
	DB08873	Boceprevir	0.997	1.000	0.840	0.913	0.915	1.000	—	—	—	—	1.000	—	—	—	—
	DB08934	Sofosbuvir	1.000	1.000	0.987	0.993	0.993	1.000	—	—	—	—	1.000	—	—	—	—
	DB09065	Cobicistat	0.993	0.972	1.000	0.986	0.981	0.986	0.000	—	0.000	—	0.990	0.000	—	0.000	—
	DB09101	Elvitegravir	0.992	0.970	1.000	0.985	0.980	0.983	0.000	—	0.000	—	0.995	0.000	—	0.000	—
	DB09102	Daclatasvir	0.999	1.000	0.946	0.972	0.972	1.000	—	—	—	—	1.000	—	—	—	—
	DB09183	Dasabuvir	0.999	1.000	0.946	0.972	0.972	1.000	—	—	—	—	1.000	—	—	—	—
	DB09297	Paritaprevir	0.999	1.000	0.946	0.972	0.972	1.000	—	—	—	—	1.000	—	—	—	—
	DB09299	Tenofovir alafenamide	0.972	0.959	0.933	0.946	0.927	0.988	0.000	—	0.000	—	0.985	0.000	—	0.000	—
validation dataset	DB00238	Nevirapine	0.989	0.000	—	0.000	—	0.994	0.988	1.000	0.994	0.987	0.995	0.000	—	0.000	—
	DB00369	Cidofovir	1.000	—	—	—	—	0.998	—	0.000	0.000	—	1.000	—	—	—	—
	DB00495	Zidovudine	0.994	0.000	—	0.000	—	0.993	0.986	1.000	0.993	0.985	0.995	0.000	—	0.000	—
	DB00625	Efavirenz	0.989	0.000	—	0.000	—	0.997	0.994	1.000	0.997	0.993	1.000	—	—	—	—
	DB00705	Delavirdine	0.991	0.000	—	0.000	—	0.998	0.996	1.000	0.998	0.996	1.000	—	—	—	—
	DB00900	Didanosine	0.988	0.000	—	0.000	—	0.993	0.988	1.000	0.994	0.987	0.982	0.000	—	0.000	—
	DB00932	Tipranavir	0.990	0.000	—	0.000	—	0.993	0.986	1.000	0.993	0.985	0.990	0.000	—	0.000	—
	DB01004	Ganciclovir	1.000	—	—	—	—	0.998	—	0.000	0.000	—	1.000	—	—	—	—
	DB06614	Peramivir	0.987	0.000	—	0.000	—	0.989	0.990	0.990	0.990	0.979	0.985	0.000	—	0.000	—
	DB09027	Ledipasvir	1.000	—	—	—	—	0.963	1.000	0.324	0.490	0.559	1.000	—	—	—	—
	DB09296	Ombitasvir	1.000	—	—	—	—	0.985	1.000	0.730	0.844	0.847	1.000	—	—	—	—
testing dataset	DB00701	Amprenavir	0.993	0.000	—	0.000	—	0.991	0.000	—	0.000	—	0.999	0.998	1.000	0.999	0.997
	DB00709	Lamivudine	0.992	0.000	—	0.000	—	0.985	0.000	—	0.000	—	0.952	0.998	0.933	0.964	0.897
	DB01072	Atazanavir	0.988	0.000	—	0.000	—	0.989	0.000	—	0.000	—	0.997	0.996	1.000	0.998	0.993
	DB01610	Valganciclovir	1.000	—	—	—	—	1.000	—	—	—	—	0.997	—	0.000	0.000	—
	DB04835	Maraviroc	0.987	0.000	—	0.000	—	0.995	0.000	—	0.000	—	0.994	0.992	1.000	0.996	0.987
	DB06290	Simeprevir	1.000	—	—	—	—	1.000	—	—	—	—	0.996	1.000	0.959	0.979	0.977
	DB08930	Dolutegravir	0.989	0.000	—	0.000	—	0.995	0.000	—	0.000	—	0.997	0.996	1.000	0.998	0.993

Also, the generally high accuracy reveals that the model effectively discriminated mismatches. High precision and recall values reveal its ability to identify and rediscover most drugs. High F1-scores and overall prediction results reflected by MCCs help address potential concerns of the imbalanced data setup. In comparing the prediction probabilities, accuracy is mostly more than precision. It ascribes to the strong ability of the model to scrutinize a drug for unmatched viral diseases.

### 3.1. *Post hoc* analysis

The meticulous refinements implemented had significant impacts in performance. A rigorous assessment, detailed in table 1, systematically scrutinizes the influence of each refinement across a number of metrics. Notably, the top row presents the performance of the complete proposed model. It serves as a reference for comparative analysis. Subsequent rows unveil nuanced variations achieved through the exclusion of specific refinements, colloquially referred to as the deep learning *tricks*. Of particular significance, pretraining with autoencoder emerges as a pivotal trick, as elucidated in the second row. While the model bereft of pretraining encoders exhibited marginally elevated AUC on the training data partition (99.9% versus 99.5%), its performance across other metrics, including accuracy and MCC, was conspicuously inferior. Similar deficiency extended to both validation and test datasets, indicating a pronounced overfitting issue. This observation agrees with the performance plot depicted in figure 2. Nonetheless, sole reliance on pretraining with autoencoder was insufficient for optimal outcomes. Additional stratagems, as delineated from the third row onwards, also contributed significantly to performance enhancement. Notably, the incorporation of self-attention mechanisms and a region proposal network also yielded substantial improvements. This underscores the critical importance of considering both local and global contextual cues, as well as the precise delineation of object bounds and objectness. All these tricks matter very much in investigating the interaction.

Anyhow, the overall best performance was achieved with a complete model based on the proposed architecture. These results demonstrate the importance of using pre-training encoders, and the various techniques to avoid over-parametrization and improve generalization.

Results from the primary analysis above indicate that Ledipasvir and Ombitasvir might share some bio-physicochemical characteristics with other drugs in the training data. Therefore, their chemical diversity should play some role in facilitating the model’s effectiveness. This section also explores the model’s effectiveness upon chemical diversity. It suggests that chemical diversity may be an important factor for this kind of analysis, but further studies with larger and more diverse datasets are needed to confirm this finding.

To optimize the datasets for model training, we need to have drugs of high chemical diversity and structural variation. However, there are not many antiviral drugs approved by the FDA, which posited constraint in the analysis. The model accuracy depends on how drug data is split. For example, if we train the model on drugs that are chemically diverse, it might perform better. On the other hand, if a data split only contains similar structures, the model might miss important features, resulting in a biased model that cannot be generalized to other possible chemical structures for a viral disease. Therefore, performance would hence deteriorate.

To test for the sensitivity to this imperfect data, I assessed the framework again with *k*-fold cross-validation on five additional datasets. In this *post hoc* analysis, the whole training process was repeated with the same model configuration, and the 58 antiviral drugs were partitioned randomly into training, validation and test data. They shall help understand the effects of chemical diversity on the model training. Table 3 shows the data split of each partition.

**Table 3.** Composition of partitions on model and cross-validation datasets. Training process of the proposed model was repeated using different partitioning of the 58 FDA-approved antiviral drugs, with five rounds of cross-validation datasets. Due to the rarity of chemical diversity and structural variation in available data, the customary deep learning practice of averaging test results over different rounds for a single performance estimate is inappropriate in this application. These cross-validation datasets demonstrate how the composition of drugs in the partitioning process (hence the chemical diversity and structural variation in the data) could affect the trained model.

dataset	model dataset	CV-dataset 1	CV-dataset 2	CV-dataset 3	CV-dataset 4	CV-dataset 5
authentic matches in training data	DB00194	DB00194	DB00194	DB00194	DB00194	DB00194
	DB00198	DB00198	DB00220	DB00198	DB00198	DB00220
	DB00220	DB00220	DB00238	DB00220	DB00220	DB00249
	DB00224	DB00249	DB00299	DB00224	DB00224	DB00369
	DB00249	DB00300	DB00300	DB00238	DB00238	DB00426
	DB00299	DB00432	DB00369	DB00299	DB00249	DB00495
	DB00300	DB00442	DB00432	DB00369	DB00299	DB00503
	DB00426	DB00478	DB00442	DB00442	DB00432	DB00529
	DB00432	DB00495	DB00478	DB00495	DB00442	DB00558
	DB00442	DB00503	DB00495	DB00503	DB00478	DB00577
	DB00478	DB00529	DB00503	DB00529	DB00503	DB00625
	DB00503	DB00558	DB00529	DB00577	DB00529	DB00649
	DB00529	DB00577	DB00577	DB00625	DB00558	DB00701
	DB00558	DB00625	DB00625	DB00649	DB00577	DB00705
	DB00577	DB00649	DB00649	DB00709	DB00625	DB00709
	DB00649	DB00701	DB00718	DB00718	DB00705	DB00718
	DB00718	DB00705	DB00787	DB00787	DB00709	DB00811
	DB00787	DB00709	DB00900	DB00879	DB00879	DB00900
	DB00811	DB00718	DB00915	DB00915	DB00915	DB00932
	DB00879	DB00787	DB00932	DB00932	DB00943	DB01004
	DB00915	DB00811	DB00943	DB00943	DB01004	DB01048
	DB00943	DB00879	DB01048	DB01004	DB01072	DB01232
	DB01048	DB00900	DB01232	DB01048	DB01232	DB01264
	DB01232	DB00932	DB01265	DB01232	DB01264	DB01319
	DB01264	DB00943	DB01319	DB01265	DB01319	DB01610
	DB01265	DB01004	DB01601	DB01601	DB01601	DB05521
	DB01319	DB01264	DB01610	DB01610	DB01610	DB06290
	DB01601	DB01319	DB04835	DB04835	DB04835	DB06414
	DB05521	DB01601	DB06290	DB05521	DB05521	DB06614
	DB06414	DB01610	DB06414	DB06414	DB06290	DB06817
	DB06817	DB05521	DB06614	DB06614	DB06614	DB08864
	DB08864	DB06290	DB06817	DB08873	DB06817	DB08873
	DB08873	DB06817	DB08873	DB08930	DB08864	DB08934
	DB08934	DB08930	DB08930	DB08934	DB08873	DB09027
	DB09065	DB08934	DB08934	DB09027	DB09027	DB09065
	DB09101	DB09065	DB09027	DB09065	DB09065	DB09102
	DB09102	DB09101	DB09065	DB09101	DB09101	DB09183
	DB09183	DB09102	DB09101	DB09102	DB09183	DB09296
	DB09297	DB09183	DB09296	DB09183	DB09296	DB09297
	DB09299	DB09296	DB09299	DB09299	DB09299	DB09299
authentic matches in validation data	DB00238	DB00238	DB00224	DB00249	DB00300	DB00198
	DB00369	DB00369	DB00249	DB00300	DB00369	DB00224
	DB00495	DB00426	DB00426	DB00432	DB00426	DB00238
	DB00625	DB01048	DB00701	DB00478	DB00495	DB00299
	DB00705	DB01072	DB00705	DB00701	DB00900	DB00787
	DB00900	DB01232	DB00709	DB00705	DB00932	DB00879
	DB00932	DB01265	DB00879	DB00811	DB01048	DB00943
	DB01004	DB06614	DB01264	DB01072	DB01265	DB01072
	DB06614	DB08873	DB05521	DB01264	DB08930	DB01601
	DB09027	DB09297	DB09102	DB08864	DB09102	DB04835
	DB09296	DB09299	DB09297	DB09297	DB09297	DB08930
authentic matches in test data	DB00701	DB00224	DB00198	DB00426	DB00649	DB00300
	DB00709	DB00299	DB00558	DB00558	DB00701	DB00432
	DB01072	DB00915	DB00811	DB00900	DB00718	DB00442
	DB01610	DB04835	DB01004	DB01319	DB00787	DB00478
	DB04835	DB06414	DB01072	DB06290	DB00811	DB00915
	DB06290	DB08864	DB08864	DB06817	DB06414	DB01265
	DB08930	DB09027	DB09183	DB09296	DB08934	DB09101

**Figure 3.**
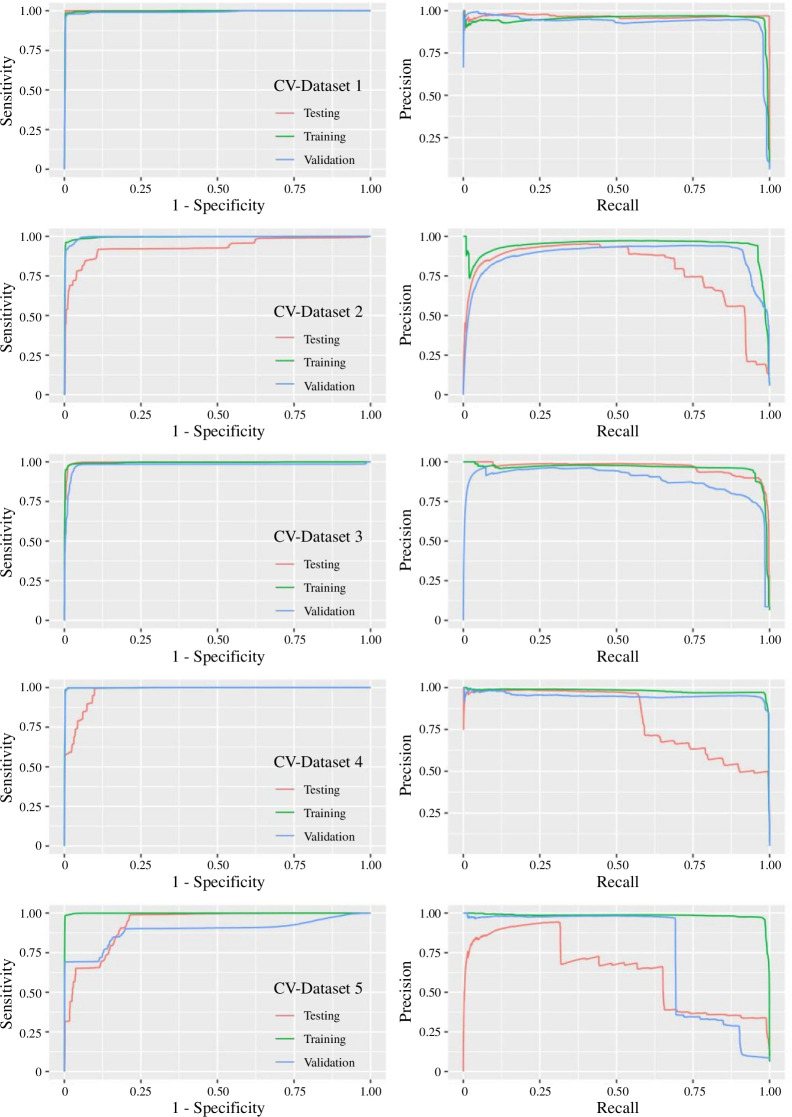
Assessing sensitivity to data with k-fold cross-validation. The sets of ROC (left) and PR curves (right) of five different cross-validation datasets demonstrate the role of chemical diversity in training the model. The purpose is to estimate the expected level of fit and errors to independent datasets. PR curves assume a threshold value that decreases from left to right. On the far left, the thresholds were so high that only a few FPs and TPs existed. While defining precision as TP / (TP + FP), a difference of 1 TP could change precision very much. In the extreme cases, the thresholds were so high that there was no TP (= 0), and the precision would be 0 (in CV-dataset 2, 3 and 5). Hence, we observe the volatility in those high-threshold regions.

The model performance on the datasets in terms of ROC and PR curves is shown in figure 3. As expected, not all the datasets could perform. The best dataset, CV-dataset 1, scored test results of 99.9% recall, 99.7% AUC, 96.1% Precision, 0.980 F1-Score and 0.978 MCC. The worst dataset, CV-dataset 5, achieved test results of 31.5% recall, 65.6% AUC, 93.8% Precision, 0.472 F1-Score and 0.521 MCC. Its trained model was overfitted to the training data. Optimizing the distribution of elements for training is critical in the partition process under the constraint of limited data, which is not unusual in medical frontiers.

**Figure 4.**
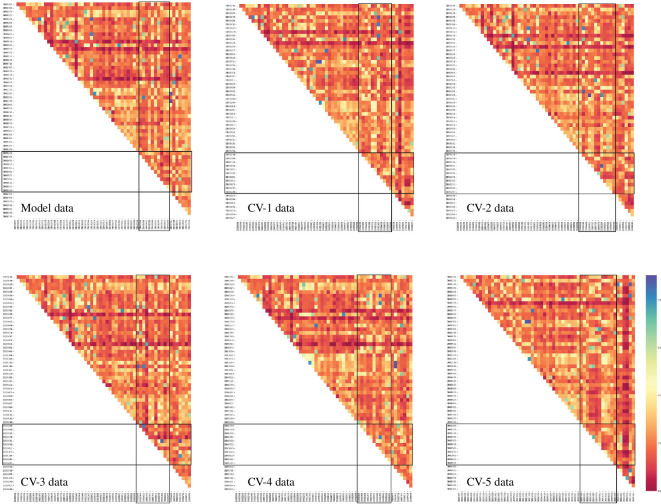
Assessing drug–drug similarity in partitioning for the model data and CV-1 to CV-5. The heatmaps exhibit the drug-drug structural similarity (or chemical diversity) of the model dataset and the five different cross-validation datasets, corresponding to table 3. Each of them shows how similar are the distributed antiviral drugs across the training, validation and testing data. A triangle shows drugs’ similarity within a partition. A rectangle shows drugs’ similarity across partitions. The purpose is to demonstrate the role of chemical diversity in model training. Hence, we observe the power of the model to learn the drugs’ properties in the context of viral diseases without using their information on structural similarity.

The potential impact of drug similarities on our model prediction performance cannot be overlooked^2^. Understanding such relationships and their therapeutic mechanisms has traditionally proven instrumental in enhancing the efficiency of new drug research, particularly valuable in drug repurposing applications [77]. To investigate whether drug similarity might influence prediction accuracy, figure 4 presents the pairwise drug similarity based on their structural makeup [78]. The heatmaps depict similarity scores ranging from 0 to 1, represented by red and blue colours, respectively. The large triangles in the top-left section of the heatmaps show the similarity of drugs in training data; they play a critical role in the training process. In the model dataset’s heatmap, some drugs exhibit significant similarity between the training and testing data, noticeable in the top-right rectangle with three blue dots. Also, there are moderately similar drugs across the training and validation data, shown in the top-centre rectangle.

However, it is important to note that high drug similarity alone does not guarantee superior prediction accuracy. This is exemplified by the CV−5 dataset’s heatmap in figure 4, where drugs also display similarity between the training and testing data (blue dots in the top-right rectangle), yet the model’s performance on this dataset is the least satisfactory. We have to investigate thoroughly the issue regarding the training processes with different datasets. Comparing the training datasets (the top-left large triangles) between CV−5 and the model data, it is evident that the model data displays greater structural dissimilarity, marked by the abundant presence of red zones. In fact, the training datasets of CV−1 and CV−3 exhibit more or less the same level of drug dissimilarity, suggesting their potential contribution to enhancing model performance, which is true in our results.

Notably, the training data associated with CV−5 stands out for having the lowest degree of drug dissimilarity. As outlined in §2, chemical diversity is essential for a model to learn. Weak drug similarity (or high chemical diversity) in the training data facilitates model training. This *post hoc* analysis emphasizes the pivotal role of chemical diversity in the training process, affirming that the model can discern and learn about the properties of drugs even in the absence of explicit structural similarity information. This underscores the model capability to understand the functional properties of drugs related to viral diseases in the latent space.

## 4. Discussion

This study aimed to overcome the challenge of limited knowledge on antiviral drugs by transforming the problem into a supervised classification model, with an unconventional data stratification. The model learned latent features from the InChI and FASTA representations and predicted the drug effectiveness without prior knowledge of the binding specificity. Its principle is partly similar to the gene set enrichment analysis in trying to extract biological insight from genomic data to identify groups of genes that share common biological functions or biological pathways [79]. However, the method presented here has merits over the conventional method by eliminating the need for explicitly identified gene sets and their expression profiles; thus, even non-coding regions could be taken into consideration in the analysis process.

From the therapeutic perspective, this study focused on the eventual outcome of a drug’s effectiveness on viral disease. The model learned important characteristics based on the InChI and FASTA representations by a deep learning technique called end-to-end learning. However, the learned latent features remain unexplained. It inherited a limitation from deep learning, that it did not show how and what specific genes are expressed, or regulated. It only predicted the eventual outcome from its learned latent knowledge, which presumably related to the common patterns of gene expression and interactions. A drug might work in various ways: say, directly interfere with the viral pathogenicity by docking on the viral genes just as theories of thermodynamics and kinetics on binding site postulate [80]; or indirectly, by modifying the precondition of the cell complex or proteins, repressing the virus–receptor interactions at the cell membranes, working on the functional properties of a virus spike protein [7], or interdicting the initiation of transcription process (or any stage of the gene expression) of the virus within a cell [81].

This study proposed a different approach from the target-based paradigm. It exploited automatic feature extraction by deep learning to avoid the difficulties of incomplete information about the actual molecular interactions, that the target-based paradigm has to cope with. Thus, this phenotypic approach could explore complex biological mechanisms that are otherwise inaccessible, due to complexities such as interferences by overlooked exons or hidden binding pockets, functions of recoded stop codons or regulators, and structural entanglement factors. Biological mechanisms were captured in the latent space. Compared to similar approaches in the literature, the network-based approach yielded around 70% accuracy for identification of well-known drug indications [49]. By using transcriptomic signatures or integrating chemical-chemical interactions and drug similarities to predict the anatomical therapeutic chemical drug class with machine learning methods, an accuracy around 75% can be obtained [82]. More recently, feature selection techniques for treatment prediction, with a random forest classifier, could yield as high as 89% in AUC [83]. Compared with these, the results of this study suggest that learned latent features with whole-genomic information were valuable for predicting drug effectiveness. However, the model could not rediscover some drugs when the number of cases was small. This is a common limitation of the deep learning approach [84]. While this approach seems feasible, it supplements the toolset in metabolomics [85] and calls for more comprehensive data collection in the research community. It also contributes to the literature on whole-genome analysis in the drug discovery context.

The results also provided some interesting insights into the drug–virus interactions. The analysis strategy did not differentiate between DNA and RNA viruses when GenBank documents RNA viruses in DNA format: the base uracil (U) is represented by thymine (T). Although such difference is obsolete after RNA synthesis, single-stranded RNA chains usually fold up into more complex shapes. Nevertheless, this did not seem to affect the latent interaction search, as the folding structures did not pose a problem in the latent space. This may also imply that the drug effectiveness might be more associated with gene expression after transcription than with direct molecular binding. This supports the idea of investigating drug working mechanisms from the gene expression perspective. It also suggests that investigating molecular pathology in the latent space is viable, given sufficient structural and genetic variations and chemical diversity. The result may extend and support rational drug design.

This study also did not consider genotype or epigenetics, which may alter virus gene expression and drug interaction. The variability includes enzymes and other intra-cellular factors, like catalysts [86], polymerases, cytochrome inhibitors or inducers [87]. Despite the significant roles of accessory proteins for transcription initiation and regulation, which might be prime antiviral drug targets [88], the current framework disregarded those transcription factors. This study only presented a simplified picture of the drug–virus interaction. However, the results are considerably higher in prediction accuracy when compared to similar approaches like the network-based approach or drug similarities as discussed above. It demonstrated that the simplification was acceptable. With more clinical data, future models may uncover more details about the interaction.

This study proposed an alternative approach to the popular target-based paradigm. It demonstrates the potential of using the latent features and interaction searches for phenotypic drug discovery. While this framework can be extended to study more complex biochemical and phenotypic processes, such as epigenetics or drug synergism, the *post hoc* analysis revealed how chemical diversity in data distribution could affect the model performance and generalization. This study thus highlights some limitations and implications of the phenotypic drug discovery approach that researchers should be aware of. Also, other deep learning techniques may also be applicable. This study informs future research on the feasibility and challenges of using deep learning for antiviral drug discovery.

## Data Availability

The datasets for training, together with the results that support the findings of this study are available at https://figshare.com/s/bd39a827c4bdf2b4a7c2 and [89]. Supplementary material is available online [90].

## Footnotes

^1^
Datasets are available at https://figshare.com/s/bd39a827c4bdf2b4a7c2.

^2^
Thanks to the two anonymous reviewers who pointed out the issues of possible drug similarities.
